# *catena*-Poly[[dibromidomercury(II)]-μ-3-(1-methyl­pyrrolidin-2-yl)pyridine-κ^2^ *N*:*N*′]

**DOI:** 10.1107/S1600536808030055

**Published:** 2008-09-24

**Authors:** Zhengjing Jiang, Guodong Tang, Yu Zhang, Jianying Zhao

**Affiliations:** a Department of Chemistry, Huaiyin Teachers College, Huai’an 223300, Jiangsu, People’s Republic of China; b Key Laboratory for Soft Chemistry and Functional Materials of the Ministry of Education, Nanjing University of Science and Technology, 200 Xiaolingwei, Nanjing 210094, Jiangsu, People’s Republic of China

## Abstract

In the title polymeric complex, [HgBr_2_(C_10_H_14_N_2_)]_*n*_, each nicotine mol­ecule is bonded to two adjacent Hg atoms, one through the pyrrolidine N atom and the other through the pyridine N atom, forming zigzag chains along [010]. The coordination around mercury is completed by two bromido ligands resulting in a distorted tetra­hedral arrangement.

## Related literature

For other nicotine complexes of copper and mercury, see: Meyer *et al.* (2006 ▶); Haendler (1990 ▶). For the isostructural dichlorido(nicotine)mercury(II) chain polymer complex, see: Udupa & Krebs (1980 ▶);
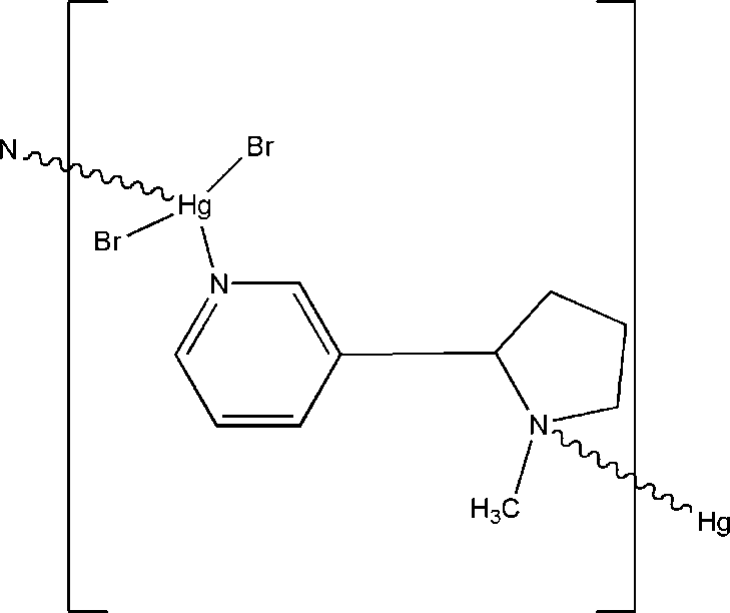


## Experimental

### 

#### Crystal data

[HgBr_2_(C_10_H_14_N_2_)]
*M* *_r_* = 522.64
Orthorhombic, 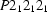

*a* = 7.6306 (9) Å
*b* = 11.2177 (14) Å
*c* = 15.443 (2) Å
*V* = 1321.9 (3) Å^3^
*Z* = 4
Mo *K*α radiation
μ = 17.66 mm^−1^
*T* = 296 (2) K
0.20 × 0.16 × 0.12 mm

#### Data collection

Bruker SMART APEXII CCD diffractometer
Absorption correction: multi-scan (*SADABS*; Bruker, 2000 ▶) *T*_min_ = 0.062, *T*_max_ = 0.153 (expected range = 0.049–0.120)
10476 measured reflections
2601 independent reflections
2137 reflections with *I* > 2σ(*I*)
*R*_int_ = 0.057

#### Refinement

*R*[*F* ^2^ > 2σ(*F* ^2^)] = 0.038
*wR*(*F* ^2^) = 0.076
*S* = 1.00
2601 reflections
137 parameters
H-atom parameters constrained
Δρ_max_ = 1.44 e Å^−3^
Δρ_min_ = −1.03 e Å^−3^
Absolute structure: Flack, (1983 ▶), 1083 Friedel pairs
Flack parameter: −0.006 (16)

### 

Data collection: *APEX2* (Bruker, 2004 ▶); cell refinement: *SAINT* (Bruker, 2004 ▶); data reduction: *SAINT*; program(s) used to solve structure: *SHELXS97* (Sheldrick, 2008 ▶); program(s) used to refine structure: *SHELXL97* (Sheldrick, 2008 ▶); molecular graphics: *SHELXL97* (Sheldrick, 2008 ▶); software used to prepare material for publication: *SHELXL97* and *PLATON* (Spek, 2003 ▶).

## Supplementary Material

Crystal structure: contains datablocks I, global. DOI: 10.1107/S1600536808030055/si2110sup1.cif

Structure factors: contains datablocks I. DOI: 10.1107/S1600536808030055/si2110Isup2.hkl

Additional supplementary materials:  crystallographic information; 3D view; checkCIF report

## Figures and Tables

**Fig. 1.**
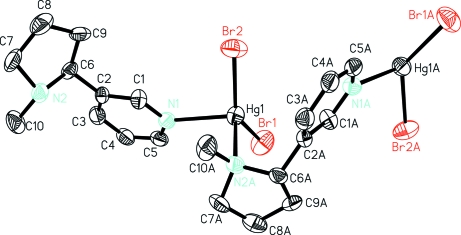
Molecular structure of the title compound, with atom labels and 50% probability displacement ellipsoids. All H atoms have been omitted. Symmetry transformations: A is -x + 1/2, -y, z + 1/2.

## supplementary crystallographic information

### Comment

Compounds containing nicotine [3-(1-methyl-2-pyrrolidinyl) pyridine] have been reported to form molecular and polynuclear complexes (Meyer *et al.*, 2006; Haendler, 1990). The crystal structure of the title compound appeared to be isostructural with the dichlorido(nicotine)mercury(II) chain polymer complex (Udupa & Krebs, 1980).

As illustrated in Fig. 1, each nicotine molecule in (I) is coordinated to two adjacent mercury atoms, one through the pyrrolidine nitrogen (Hg—N 2.400 (8) Å) and the other through the pyridine nitrogen (Hg—N 2.460 (8) Å), forming zigzag polymeric chains along the *b* axis. The coordination around mercury is completed by two bromine ligands (Hg—Br 2.4760 (12) and 2.5034 (12) Å), resulting in a distorted tetrahedral arrangement. In addition, the absolut configurations of C6 and N2 can be given as *S* (*S*-nicotine was used as a starting material). No notable interactions were found between polymeric chains.

### Experimental

HgBr_2_ (360 mg,1 mmol) was added to a solution of 4-cyanopyridine (104 mg,1 mmol) in dmf (5 ml). The resulting mixture was stirred for about 10 min after which an white precipitate formed. *S*-Nicotine (3 ml) was then added dropwise to the reaction mixture and stirring was continued, during which time the precipitate changed its colour, giving a flesh colored precipitate. The precipitate was washed with ethanol and vacuum dried. Yield: 0.324 g, 62% (based on HgBr_2_used). The compound (100 mg) was dissolved in dmf (5 ml), the resulting solution filtered and the light-yellow filtrate transfered into a test tube and *i*-PrOH (10 ml) was carefully laid on the surface of the filtrate. Light-yellow block crystals were obtained after 15 days. Analysis: Found: C 23.12, H 2.82, N 5.26%; Calculated for C_10_H_14_HgBr_2_N_2_: C 22.98, H 2.70, N 5.36%.

### Refinement

H atoms were positioned geometrically and refined using a riding model, with C—H = 0.93 - 0.98 Å and with *U*_iso_(H) = 1.2 (1.5 for methyl groups) times *U*_eq_(C). The absolute structure parameter *x* (Flack, 1983) was refined to -0.006 (16) using 1083 measured Friedel pairs.

### Crystal data

**Table:**

[HgBr_2_(C_10_H_14_N_2_)]	*F*(000) = 952
*M**_r_* = 522.64	*D*_x_ = 2.626 Mg m^−^^3^
Orthorhombic, *P*2_1_2_1_2_1_	Mo *K*α radiation, λ = 0.71073 Å
Hall symbol: P 2ac 2ab	Cell parameters from 3183 reflections
*a* = 7.6306 (9) Å	θ = 4.5–43.1°
*b* = 11.2177 (14) Å	µ = 17.66 mm^−^^1^
*c* = 15.443 (2) Å	*T* = 296 K
*V* = 1321.9 (3) Å^3^	Block, light yellow
*Z* = 4	0.20 × 0.16 × 0.12 mm

### Data collection

**Table:**

Bruker SMART APEXII CCD diffractometer	2601 independent reflections
Radiation source: fine-focus sealed tube	2137 reflections with *I* > 2σ(*I*)
graphite	*R*_int_ = 0.057
φ and ω scans	θ_max_ = 26.0°, θ_min_ = 2.2°
Absorption correction: multi-scan (SADABS; Bruker, 2000)	*h* = −8→9
*T*_min_ = 0.062, *T*_max_ = 0.153	*k* = −13→13
10476 measured reflections	*l* = −19→16

### Refinement

**Table:**

Refinement on *F*^2^	Secondary atom site location: difference Fourier map
Least-squares matrix: full	Hydrogen site location: inferred from neighbouring sites
*R*[*F*^2^ > 2σ(*F*^2^)] = 0.039	H-atom parameters constrained
*wR*(*F*^2^) = 0.076	*w* = 1/[σ^2^(*F*_o_^2^) + (0.0314*P*)^2^] where *P* = (*F*_o_^2^ + 2*F*_c_^2^)/3
*S* = 1.00	(Δ/σ)_max_ = 0.001
2601 reflections	Δρ_max_ = 1.44 e Å^−^^3^
137 parameters	Δρ_min_ = −1.03 e Å^−^^3^
0 restraints	Absolute structure: Flack, (1983), 1083 Friedel pairs
Primary atom site location: structure-invariant direct methods	Flack parameter: −0.006 (16)

### Special details

**Table:**

Geometry. All e.s.d.'s (except the e.s.d. in the dihedral angle between two l.s. planes) are estimated using the full covariance matrix. The cell e.s.d.'s are taken into account individually in the estimation of e.s.d.'s in distances, angles and torsion angles; correlations between e.s.d.'s in cell parameters are only used when they are defined by crystal symmetry. An approximate (isotropic) treatment of cell e.s.d.'s is used for estimating e.s.d.'s involving l.s. planes.
Refinement. Refinement of *F*^2^ against ALL reflections. The weighted *R*-factor *wR* and goodness of fit *S* are based on *F*^2^, conventional *R*-factors *R* are based on *F*, with *F* set to zero for negative *F*^2^. The threshold expression of *F*^2^ > σ(*F*^2^) is used only for calculating *R*-factors(gt) *etc*. and is not relevant to the choice of reflections for refinement. *R*-factors based on *F*^2^ are statistically about twice as large as those based on *F*, and *R*- factors based on ALL data will be even larger.

### Fractional atomic coordinates and isotropic or equivalent isotropic displacement parameters (Å^2^)

**Table:**

	*x*	*y*	*z*	*U*_iso_*/*U*_eq_	
Br1	0.09573 (17)	1.16819 (12)	0.93970 (8)	0.0688 (4)	
Br2	0.15737 (16)	1.07721 (14)	0.64794 (8)	0.0622 (4)	
C1	0.2197 (13)	0.7896 (8)	0.7769 (6)	0.035 (2)	
H1	0.1843	0.8032	0.7201	0.042*	
C2	0.3218 (11)	0.6891 (8)	0.7935 (6)	0.028 (2)	
C3	0.3679 (13)	0.6710 (9)	0.8800 (7)	0.044 (3)	
H3	0.4355	0.6053	0.8952	0.052*	
C4	0.3143 (14)	0.7495 (9)	0.9428 (7)	0.045 (3)	
H4	0.3438	0.7373	1.0005	0.054*	
C5	0.2161 (14)	0.8467 (9)	0.9184 (6)	0.040 (3)	
H5	0.1806	0.9003	0.9609	0.048*	
C6	0.3777 (12)	0.6034 (8)	0.7256 (6)	0.034 (2)	
H6	0.4914	0.5703	0.7427	0.041*	
C7	0.3129 (14)	0.4423 (10)	0.6345 (7)	0.055 (3)	
H7A	0.2193	0.3962	0.6084	0.066*	
H7B	0.4103	0.3896	0.6470	0.066*	
C8	0.3698 (16)	0.5418 (10)	0.5746 (7)	0.058 (3)	
H8A	0.2811	0.5570	0.5310	0.070*	
H8B	0.4789	0.5216	0.5459	0.070*	
C9	0.3936 (14)	0.6494 (9)	0.6325 (6)	0.047 (3)	
H9A	0.3041	0.7086	0.6207	0.057*	
H9B	0.5078	0.6852	0.6231	0.057*	
C10	0.2521 (15)	0.4153 (9)	0.7882 (7)	0.054 (3)	
H10A	0.1949	0.3428	0.7712	0.081*	
H10B	0.1910	0.4499	0.8365	0.081*	
H10C	0.3708	0.3983	0.8047	0.081*	
Hg1	0.04788 (5)	1.06376 (4)	0.80044 (3)	0.04142 (13)	
N1	0.1699 (10)	0.8672 (7)	0.8373 (5)	0.036 (2)	
N2	0.2514 (11)	0.5010 (7)	0.7140 (5)	0.040 (2)	

### Atomic displacement parameters (Å^2^)

**Table:**

	*U*^11^	*U*^22^	*U*^33^	*U*^12^	*U*^13^	*U*^23^
Br1	0.0714 (10)	0.0714 (9)	0.0635 (8)	0.0005 (7)	−0.0093 (6)	−0.0279 (7)
Br2	0.0525 (7)	0.0861 (10)	0.0481 (7)	0.0000 (7)	0.0091 (5)	0.0157 (7)
C1	0.043 (6)	0.033 (6)	0.029 (6)	0.005 (5)	−0.011 (4)	−0.001 (4)
C2	0.026 (5)	0.025 (5)	0.032 (5)	0.002 (4)	−0.005 (4)	−0.001 (4)
C3	0.037 (6)	0.034 (6)	0.060 (7)	0.000 (5)	0.000 (5)	0.004 (5)
C4	0.059 (8)	0.038 (6)	0.037 (6)	−0.015 (5)	0.006 (5)	0.000 (5)
C5	0.050 (7)	0.037 (6)	0.032 (6)	−0.011 (5)	0.008 (5)	−0.001 (5)
C6	0.023 (5)	0.037 (6)	0.044 (6)	0.005 (4)	0.001 (4)	−0.009 (4)
C7	0.051 (7)	0.042 (7)	0.072 (8)	0.012 (6)	0.014 (6)	−0.020 (7)
C8	0.068 (8)	0.066 (9)	0.041 (7)	0.010 (7)	0.009 (6)	−0.015 (6)
C9	0.046 (7)	0.052 (7)	0.044 (7)	−0.011 (5)	0.023 (5)	−0.003 (5)
C10	0.052 (7)	0.039 (7)	0.072 (8)	0.007 (5)	−0.007 (6)	0.021 (6)
Hg1	0.0416 (2)	0.0400 (2)	0.0427 (2)	0.0040 (2)	−0.0016 (2)	−0.0011 (2)
N1	0.038 (5)	0.037 (5)	0.032 (5)	0.002 (4)	0.000 (4)	0.003 (4)
N2	0.030 (4)	0.035 (5)	0.054 (6)	0.006 (4)	0.001 (4)	−0.002 (4)

### Geometric parameters (Å, °)

**Table:**

Br1—Hg1	2.4760 (12)	C7—N2	1.470 (12)
Br2—Hg1	2.5034 (12)	C7—C8	1.512 (15)
C1—N1	1.332 (11)	C7—H7A	0.9700
C1—C2	1.394 (12)	C7—H7B	0.9700
C1—H1	0.9300	C8—C9	1.512 (14)
C2—C3	1.396 (14)	C8—H8A	0.9700
C2—C6	1.485 (12)	C8—H8B	0.9700
C3—C4	1.372 (14)	C9—H9A	0.9700
C3—H3	0.9300	C9—H9B	0.9700
C4—C5	1.376 (14)	C10—N2	1.496 (12)
C4—H4	0.9300	C10—H10A	0.9600
C5—N1	1.321 (12)	C10—H10B	0.9600
C5—H5	0.9300	C10—H10C	0.9600
C6—N2	1.510 (12)	Hg1—N2^i^	2.400 (8)
C6—C9	1.532 (13)	Hg1—N1	2.460 (8)
C6—H6	0.9800	N2—Hg1^ii^	2.400 (8)
			
N1—C1—C2	124.0 (9)	C7—C8—H8B	110.7
N1—C1—H1	118.0	C9—C8—H8B	110.7
C2—C1—H1	118.0	H8A—C8—H8B	108.8
C1—C2—C3	115.8 (9)	C8—C9—C6	106.0 (8)
C1—C2—C6	123.6 (9)	C8—C9—H9A	110.5
C3—C2—C6	120.6 (8)	C6—C9—H9A	110.5
C4—C3—C2	120.5 (10)	C8—C9—H9B	110.5
C4—C3—H3	119.7	C6—C9—H9B	110.5
C2—C3—H3	119.7	H9A—C9—H9B	108.7
C3—C4—C5	118.5 (10)	N2—C10—H10A	109.5
C3—C4—H4	120.7	N2—C10—H10B	109.5
C5—C4—H4	120.7	H10A—C10—H10B	109.5
N1—C5—C4	122.9 (10)	N2—C10—H10C	109.5
N1—C5—H5	118.6	H10A—C10—H10C	109.5
C4—C5—H5	118.6	H10B—C10—H10C	109.5
C2—C6—N2	113.1 (8)	N2^i^—Hg1—N1	96.8 (3)
C2—C6—C9	117.9 (8)	N2^i^—Hg1—Br1	111.1 (2)
N2—C6—C9	101.3 (7)	N1—Hg1—Br1	99.6 (2)
C2—C6—H6	108.0	N2^i^—Hg1—Br2	104.3 (2)
N2—C6—H6	108.0	N1—Hg1—Br2	98.36 (19)
C9—C6—H6	108.0	Br1—Hg1—Br2	137.68 (5)
N2—C7—C8	105.8 (8)	C5—N1—C1	118.3 (8)
N2—C7—H7A	110.6	C5—N1—Hg1	118.5 (7)
C8—C7—H7A	110.6	C1—N1—Hg1	122.2 (6)
N2—C7—H7B	110.6	C7—N2—C10	110.6 (8)
C8—C7—H7B	110.6	C7—N2—C6	103.7 (8)
H7A—C7—H7B	108.7	C10—N2—C6	113.3 (8)
C7—C8—C9	105.2 (8)	C7—N2—Hg1^ii^	110.9 (6)
C7—C8—H8A	110.7	C10—N2—Hg1^ii^	105.2 (6)
C9—C8—H8A	110.7	C6—N2—Hg1^ii^	113.3 (6)

Symmetry codes: (i) −*x*, *y*+1/2, −*z*+3/2; (ii) −*x*, *y*−1/2, −*z*+3/2.
